# Genomic mechanisms of immune evasion in colorectal cancer: from discovery to clinical practice

**DOI:** 10.18632/oncotarget.26105

**Published:** 2018-09-18

**Authors:** Catherine S. Grasso, Marios Giannakis

**Affiliations:** Marios Giannakis: Department of Medical Oncology, Dana-Farber Cancer Institute and Harvard Medical School, Boston, Massachusetts, USA; Broad Institute of MIT and Harvard, Cambridge, Massachusetts, USA

**Keywords:** colorectal cancer, immunotherapy, immune evasion, genomics

Immune checkpoint inhibitors have revolutionized the landscape of cancer therapies [1]. In colorectal cancer (CRC), the immune contexture (type, density and location of immune cells) has been shown to have prognostic significance [2], with a recent international study demonstrating the implementation and reproducibility of the “Immunoscore” as a prognostic biomarker in this disease [3]. Despite this known relevance of tumor immunity in CRC however, immune checkpoint blockade has yet to make a substantial impact on CRC treatment. Specifically, immune checkpoint inhibitors are so far effective in the microsatellite-instability high (MSI-High) tumors [4, 5], which only represent approximately 4% of all metastatic CRC. In these instances, MSI-High status correlates with elevated tumor neoantigen loads, which in turn correlate with immune response [6]. Even in this scenario however, the response rates of MSI-High tumors to immune checkpoint blockade are only 40-50%. In addition, the more common microsatellite stable CRCs, which represent the majority of metastatic colorectal tumors, are resistant to immunotherapy [4], with the underlying mechanisms not understood.

It is known, nevertheless, that CRC is a molecularly heterogeneous disease with distinct genomic drivers (e.g. *APC, RNF43, KRAS, BRAF, PIK3CA*) [6, 7] and transcriptional subtypes [8]. Investigating the association between specific molecular events in CRC and anti-tumor immunity requires the comprehensive genomic, transcriptional and pathologic characterization of the tumor-immune microenvironment in large multi-dimensional data-sets, followed by integrative studies powered to detect such associations. In our recent study [9], we combined 592 CRCs from The Cancer Genome Atlas with available whole-exome sequencing (WES), whole transcriptome (RNA-Seq) and methylation data with 619 CRCs from two prospective cohort studies with WES and immunohistochemistry (IHC) data. With a total set of over 1,200 primary tumors (including 179 MSI-High ones), we redefined genes that are significantly mutated in CRC and demonstrated an enrichment among these cancer drivers of WNT-signaling and immune-modulating genes. Mutations in the latter, which included genes of the antigen-presentation machinery (APM), were particularly enriched among MSI-High tumors, which are under immune selection pressure due to their immunogenicity. In addition, and to further support the functional relevance of these molecular events, we demonstrated biallelic inactivation and reduced expression of APM genes such as *HLA* and *beta-2 microglobulin* (*B2m*) in MSI-High colorectal tumors that in many cases involved a second-hit through copy number alterations. Inactivating mutations in *B2m* alone were present in more than 10% of MSI-High CRCs. We also discovered mutations in *NLRC5* and *RFX5*, which cooperate to induce transcription of MHC and other APM genes [10]. Such immune editing events may underlie the resistance of some MSI-High tumors to immune-checkpoint blockade consistent with reports of intrinsic and acquired resistance to immune checkpoint inhibition in melanoma [11, 12]. Given the tissue-agnostic FDA approval of pembrolizumab for all refractory MSI-High tumors and nivolumab for MSI-High CRCs, it will be interesting to correlate the presence of such molecular events with response to therapy in a systematic way. Such investigations may improve patient selection but also lead to novel immunotherapeutic approaches in patients with lost HLA expression; given the known inhibitory effect of HLA Class I proteins on Natural Killer (NK) cells [13], NK-based immunotherapies could offer an attractive option for these anti-PD1 resistant MSI-High patients.

**Figure 1:**
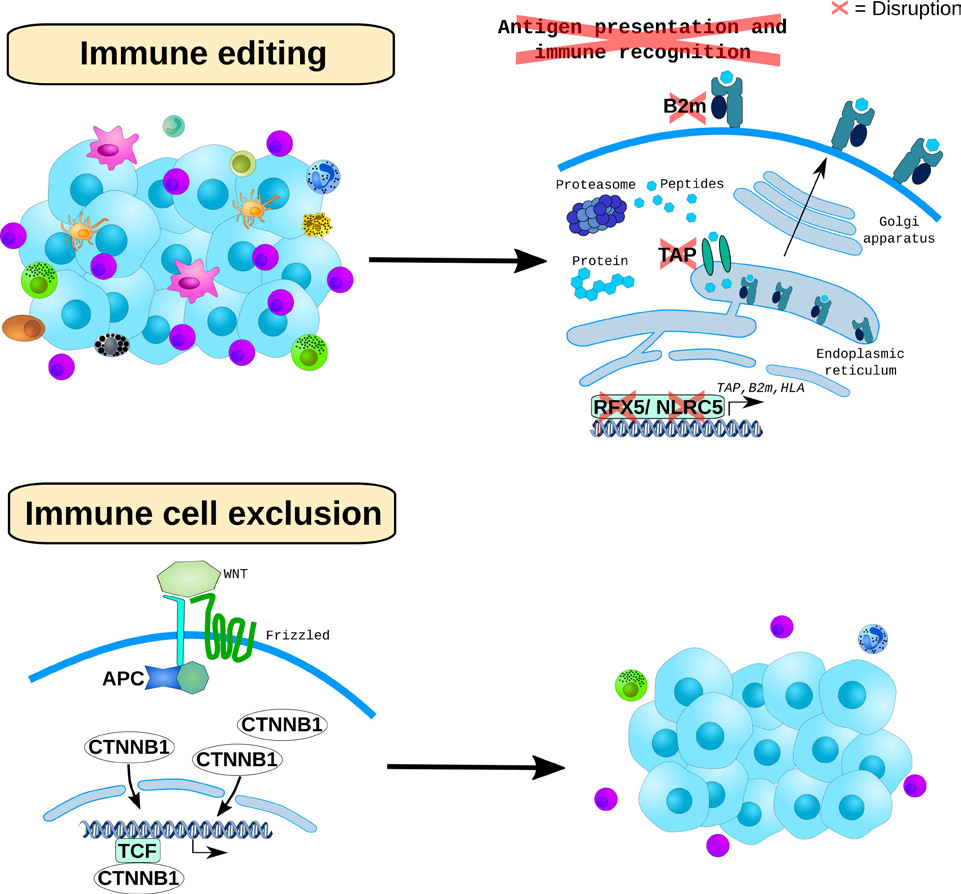
Mechanisms of immune evasion in CRC. Top panel MSI-High CRCs, that are under immune pressure, positively select mutations in APM genes, including transcriptional regulators of this pathway and *B2m*. **Bottom panel:** CRCs exclude an effective immune infiltrate through genetically and epigenetically activating WNT-signaling.

On the other hand, for most colorectal tumors including the non-MSI-High ones, additional immunotherapeutic approaches are sorely needed. Leveraging RNA-Seq and IHC data, we were able to demonstrate a recurrent inverse correlation of WNT-signaling activation, as measured by WNT-pathway transcripts and nuclear beta-catenin expression, to effector and antigen-experienced tumor-infiltrating lymphocytes, indicating the putative role of this pathway as a driver for immune checkpoint resistance in CRC [9]. We showed that underlying this association were genetic events such as biallelic *APC* inactivating mutations but also epigenetic mechanisms including hypermethylation of the *AXIN2* (a WNT target gene) super-enhancer. This role of WNT-signaling in immune cell exclusion is consistent with work in melanoma [14] and, given that this is the major and most frequently altered pathway in CRC, has implications for novel combination immunotherapy trials in this disease. WNT-signaling inhibitors such as Porcupine inhibitors are being combined with PD1 checkpoint inhibition in ongoing clinical trials and this could be a relevant strategy for a subset of colorectal tumors. Moreover, these and additional WNT-targeting compounds could find a relevant role as immunomodulators in less toxic schedules or doses compared to those needed to achieve a cell-autonomous anti-tumor response. More generally, our work demonstrates the utility of large-scale, comprehensive analyses of both tumors and their microenvironments in elucidating mechanisms of immune editing and immune cell exclusion (Figure 1) that can provide insights for novel clinical trials. High-throughput, innovative technologies such as multiplexed / multispectral imaging, mass cytometry and single cell RNA-Seq analyses combined with the right clinical cohorts and questions can further accelerate our understanding of anti-tumor immunity and translate into better treatments for cancer patients.
